# Multi-resistant Escherichia coli and mycotic aneurysm: two case reports

**DOI:** 10.1186/1752-1947-3-6453

**Published:** 2009-03-10

**Authors:** John F McCann, Azhar Fareed, Sukanya Reddy, John Cheesbrough, Neil Woodford, Sally Lau

**Affiliations:** 1 Department of Medicine for the Elderly, Lancashire Teaching Hospitals NHS Foundation Trust, Preston, PR2 9HT, UK; 2 Department of Microbiology, Lancashire Teaching Hospitals NHS Foundation Trust, Preston, PR2 9HT, UK; 3 Centre for Infections, Health Protection Agency, London, NW9 5EQ; 4 Division of Regenerative Medicine, University of Manchester, Manchester Royal Infirmary, Manchester, M13 9WZ

## Abstract

**Introduction:**

Mycotic aneurysms account for a small proportion of all aneurysms. *Escherichia coli* a gram-negative organism, is recognised as a rare cause of aortic aneurysm. We report two cases of mycotic aneurysm caused by the same strain of multi-resistant *Escherichia coli*. The purpose of this case report is to highlight the possibility that this strain may be associated with an increased risk of endovascular infection especially in extra-aortic sites. These aneurysms can be difficult to detect and can have serious consequences.

**Case presentation:**

In case one, the patient presented with symptoms and signs of septicaemia secondary to a urinary tract infection. Despite adequate treatment the patient continued with pyrexia and raised inflammatory markers, therefore a series of CT scans of the abdomen and thorax were performed, which revealed two intra-thoracic pseudo-aneurysms with associated haematomas. In case two, the patient also developed *Escherichia coli* septicaemia. On day 44 he developed a swelling on the right side of his neck. An ultrasound scan showed a pseudoaneurysm of the right common carotid artery.

**Conclusions:**

Whilst a case report cannot prove that a heightened risk exists, we suggest that it is an area worthy of further surveillance. We recommend when older patients with atheromatosis develop prolonged *Escherichia coli* septicaemia, the possibility of an infected aneurysm should be borne in mind.

## Introduction

Bloodstream infections with *Escherichia coli* (*E. coli*) in adults are most often related to underlying infection of the urinary or bilary tract or other intra-abdominal focal infections such as pericolic abscess. Metastatic blood borne infections with *E. coli* that have been reported are discitis, vertebral osteomyelitis and joint space infection [1,2]. However, endocarditis and other endovascular infections are rare [3,4].

Since 2003, strains of *E. coli* producing extended spectrum Β-lactamases (ESBLs), particularly CTX-M-15, have become increasingly common [5]. ESBLs confer resistance to third- and subsequent- generation cephalosporins. Subsequently, producer strains are often also resistant to fluoroquinolones and trimethoprim. The population of *E. coli* with CTX-M enzymes in the UK is partly clonal and one strain, "A", is particularly widespread, being dominant in some areas. Isolates of strain A that also possess an acquired AmpC Β-lactamase have recently been reported, predominantly from the North-West of England [6]. The AmpC enzyme enhances resistance to cefoxitin and to Β-lactamase inhibitor combinations, further reducing the available treatment options.

We recently encountered two unconnected cases of mycotic aneurysm caused by isolates of this multi-resistant *E. coli* strain; both produced the CTX-M and Amp C Β-lactamase. The purpose of this report is to alert clinicians to the possibility that this strain may be associated with an increased risk of endovascular infection, particularly in extra-aortic sites, which can be difficult to detect and can have serious consequences.

## Case presentation

### Case 1

A 69-year-old man was admitted in August 2005, following a six hour history of suprapubic pain, fever and rigors. He had a long term catheter for a prostate problem and his past history included a myocardial infarction in 2001, followed by a series of small strokes. As a result of these, he had a mild residual left hemiparesis and had received a stent to his right carotid artery. On examination he was pyrexial (39.2°C) with otherwise normal vital signs and no new findings. Investigations showed a low white cell count (2.5 × 10^9^/litre) and raised inflammatory markers, with an ESR of 84 mm/hr and C-reactive protein of 176 mg/l. Chest X- ray revealed no abnormalities of clinical significance.

Cultures of blood and urine grew *E. coli* resistant to all cephalosporins and fluoroquinolones but susceptible to meropenem. A diagnosis of septicaemia originating from a urinary tract infection was made. Intravenous meropenem 1 gm three times a day was commenced. However the patient continued to have raised inflammatory markers with intermittent pyrexia and meropenem was stopped after 14 days. Three sets of blood cultures taken 7 days later all grew *E. coli* with the same antibiogram as found previously. Meropenem was recommenced at the same dose.

**Figure 1:**
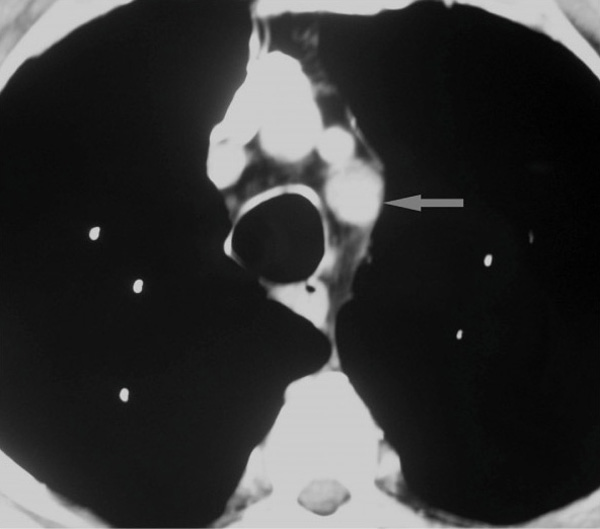
**First scan showing only mild dilation of left subclavian artery**.

**Figure 2:**
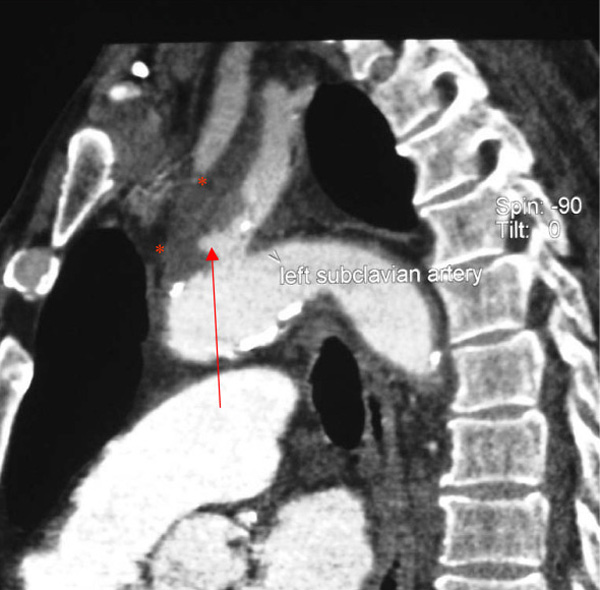
Second scan showing new mycotic aneurysms and thrombus**.

A computed tomography (CT) scan was requested to explore the possibility of lymphoma or a deep abscess. This showed a small abdominal aortic aneurysm and a mild dilatation of the left subclavian artery (Figure 1). A further scan was performed 2 weeks later to exclude leakage from the abdominal aneurysm. Whilst this possibility was discounted, two new intra-thoracic pseudo-aneurysms and associated haematomas were found; one was arising at the anterior aspect of the origin of the left subclavian artery and the second in the descending thoracic aorta (Figure 2).

**Figure 3:**
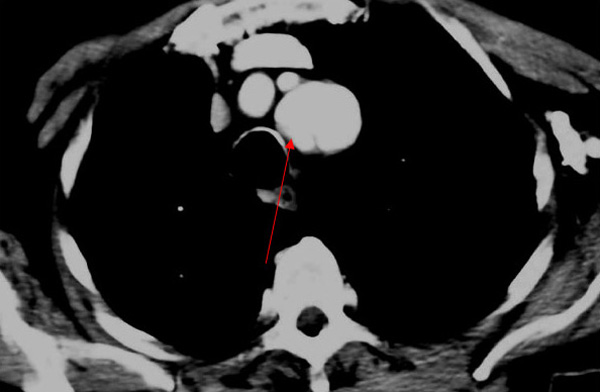
**Third scan (recuperative phase) showing reduced size of left subclavian artery aneurysm and clearing of thrombus**.

The patient was transferred to a cardiothoracic surgery centre but intervention was not advised due to his vascular co-morbidities. Instead he was returned to our care for conservative management and continuation of his antibiotics. His fever and inflammatory markers gradually settled to normal. A third scan (Figure 3) was performed after 4 weeks of consistent control over the infection. The rationale was to seek any resolution of the mycotic aneurysm as a prelude to withdrawing antibiotics. As can be seen from these final images, considerable resolution of the pseudo-aneurysms and haematomas had occurred. After a further check that blood cultures and inflammatory markers had normalised, intravenous meropenem was stopped after a total of 16 weeks. No further pyrexia occurred, and the patient remained well 3 months later.

### Case 2

An 80-year-old man was admitted in November 2004 with a 3-day history of malaise, anorexia and reduced mobility. His past history included three strokes affecting his left side. He also had a long term urinary catheter for prostatic disease. A routine catheter specimen of urine (CSU) taken three weeks prior to admission grew mixed coliform bacteria susceptible to several agents. This was not considered to be clinically significant.

On admission his vital signs were initially satisfactory and physical examination did not reveal any new abnormality. A chest X-ray showed bilateral hilar lymphadenopathy, with no clear cause. On the second day after admission, he developed transient pyrexia of 38°C. A CSU again grew coliform susceptible only to mecillinam, meropenem and gentamicin. No treatment was given.

On the 19^th^ day pyrexia (38.1°C) recurred and oral trimethoprim 200 mg twice a day was commenced for presumed urinary infection. This was changed to intravenous gentamicin 300 mg once a day after blood cultures yielded *E. coli*, susceptible only to gentamicin and meropenem. Gentamicin was stopped after a week but pyrexia recurred the following day. A diagnosis of chest infection was made and further blood cultures were taken. *E. coli* with a similar resistance profile was isolated and the patient was commenced on intravenous meropenem 500 mg, three times daily.

At day 44, he developed a swelling on the right side of his neck. An ultrasound scan showed this to be a pseudoaneurysm of the right common carotid artery. Meropenem was increased to 1 g, three times daily and gentamicin was added. In spite of apparently responding to this regimen, with the CRP falling from 203 to 23 mg/l, the patient died on day 69 after contracting aspiration pneumonia.

### Microbiological typing

The isolates from both cases were sent to the reference laboratory for molecular typing and characterisation of their Β-lactamases. Both belonged to UK epidemic *E. coli* strain A, which produces CTX-M-15 ESBL [5]. They also produced a CIT-type acquired CMY-23 AmpC Β-lactamase.

Multi Locus Sequence Typing (MLST) showed that these two isolates belonged to a genetic lineage ST 131, which has been identified as an important uro-pathogenic strain often with multiple antibiotic resistances (personal communication S.Lau).

## Discussion

Mycotic aneurysms (also known as infected or pseudoaneurysms) account for only 2.5% of all aneurysms and 1.8% of all thoracoabdominal aortic aneurysms [7,8]. Early diagnosis is important since, without medical and often surgical management, catastrophic haemorrhage or uncontrolled sepsis may occur. With the decline in bacterial endocarditis, most infected aneurysms now tend to affect the aorta and the more proximal arteries. The spectrum of causative bacteria has shifted from gram-positive to gram-negative and in one series, these latter accounted for 45% of all infected thoracoabdominal aortic aneurysms, with *E. coli* being second in importance only to *Salmonella*[9].

Increasing attention has been drawn to the link between urological processes and infected aneurysms, arising by dissemination of organisms originating from urinary tract infections with overspill to the blood, facilitated by instrumentation and indwelling catheters [10-12]. One study reports increasing age and atherosclerotic problems as risk factors [10].

Our cases featuring older atheromatous patients with urinary infection, thus far conformed to these general patterns. The new issues that we wish to underline are that multi-resistant *E. coli* have not previously been mentioned specifically in the context of infected aneurysms. Further, in both our cases, the same clone of *E. coli* was involved and the patients shared the somewhat unusual feature of developing non-aortic infected aneurysms (case 1, subclavian artery; case 2, internal carotid). We therefore speculate that either as a result of the prolonged septicaemia or a specific difference in pathogenesis, non-aortic aneurysms are more likely in this situation.

Finally, there is a recognised association between multi-resistance and virulence in other Enterobacteriaceae such as *Salmonella typhimurium* DT104. A similar linkage may operate with the clones (for example, strain A) of this multi resistant *E. coli* lineage ST131 now spreading in the UK.

## Conclusion

Whilst a case report cannot prove that a heightened risk exists, we suggest that it is an area worthy of further surveillance. We recommend when older patients with atheromatosis develop prolonged *E. coli* septicaemia, the possibility of infected aneurysm should be borne in mind. Since surgery may be precluded by co-morbidities, prompt diagnosis and prolonged antibiotic therapy is crucial.

## Abbreviations

*E.coli*: *Escherichia coli*; ESBLs: extended spectrum Β-lactamases.

## Consent

Written informed consent was obtained from the patient in case one for publication of this case report. We were unable to contact any family members of the patient presented in case two, despite our best efforts. We believe this case report holds a worthwhile clinical lesson, which could not be effectively communicated in any other way. We would not expect the family to object to publication. A copy of the written consent is available for review by the Editor-in-Chief of this journal.

## Competing interests

The authors declare that they have no competing interests.

## Authors' contributions

JFM was responsible for coordinating the study and drafting the manuscript. AF summarised the two case studies and helped to draft the manuscript. JC and SR, along with collaboration from NW and SL, assisted in identifying the strains and providing important information on the microbiological aspects of this case study.
